# Emphasis on Icosapent Ethyl for Cardiovascular Risk Reduction: A Systematic Review

**DOI:** 10.7759/cureus.32346

**Published:** 2022-12-09

**Authors:** Bansi Sutariya, Diana M Montenegro, Michael Chukwu, Paghunda Ehsan, Rawia N Aburumman, Shivani Ishwarya Muthanna, Swathi Radhakrishnan Menon, Vruti Vithani, Sai Sri Penumetcha

**Affiliations:** 1 Internal Medicine, California Institute of Behavioral Neurosciences & Psychology, Fairfield, USA; 2 General Medicine, Government Medical College, Surat, IND; 3 General Surgery, California Institute of Behavioral Neurosciences & Psychology, Fairfield, USA; 4 General Surgery, Pilgrim Hospital, Boston, GBR; 5 Research, California Institute of Behavioral Neurosciences & Psychology, Fairfield, USA; 6 Internal Medicine, Hayatabad Medical Complex Peshawar, Peshawar, PAK; 7 Internal Medicine, Mu'tah University, Amman, JOR; 8 Internal Medicine, Vydehi Institute of Medical Sciences and Research Centre, Bangalore, IND; 9 Pediatrics, California Institute of Behavioral Neurosciences & Psychology, Fairfield, USA; 10 General Medicine, California Institute of Behavioral Neurosciences & Psychology, Fairfield, USA; 11 General Medicine, Chalmeda Anand Rao Institute of Medical Sciences, Karimnagar, IND

**Keywords:** cardiovascular risk reduction, coronary artery disease, eicosapentaenoic acid (epa), vascepa, icosapent ethyl

## Abstract

Despite the widespread use of lipid-lowering agents such as statins, cardiovascular disease (CVD) remains the leading cause of mortality worldwide. Icosapent ethyl (IPE) (Vascepa), an ethyl ester of the omega-3 polyunsaturated fatty acid eicosapentaenoic acid (EPA), has gained widespread popularity as an adjunctive agent that targets multiple and additional mechanisms linked to the incidence of cardiovascular (CV) events and the causative pathway of atherosclerosis. The Preferred Reporting Items for Systematic Reviews and Meta-Analyses (PRISMA) 2020 standards were used to conduct this systematic review. In this review, we assessed various studies from PubMed, PubMed Central (PMC), and Google Scholar to evaluate the mechanisms of action and beneficial effects of IPE in the reduction of CVD outcomes. The Reduction of Cardiovascular Events with Icosapent Ethyl-Intervention Trial (REDUCE-IT) has demonstrated a significant reduction in CV mortality with 4 g/day IPE as compared to placebo. All other trials and observational studies have supported the role of Vascepa in hypertriglyceridemia and CV risk reduction. In conclusion, the use of IPE has been shown to significantly reduce triglyceride levels and reduce CV risks in patients receiving optimal statin therapy.

## Introduction and background

According to the 2017 Global Burden of Diseases, Injuries, and Risk Factors Study, atherosclerotic cardiovascular disease (ASCVD) remains the leading cause of mortality worldwide and poses a significant danger to global health [1,2]. Cardiovascular disease was responsible for over 17.8 million deaths in 2017, and by 2030, that number is anticipated to rise to 22.2 million [3]. An estimated 18.2 million Americans over the age of 20 have coronary artery disease, and 7.0 million have had a stroke [4]. While the widespread use of statins in primary and secondary prevention has greatly improved cardiovascular (CV) outcomes, a substantial residual CV risk remains elevated despite such evidence-based lipoprotein lowering treatment and may require additional therapy to lower this risk [3,5]. Several studies have demonstrated a causal link between high triglyceride (TG) levels and residual CV risks [6-9]. Prior medications targeting TG levels or high-density lipoprotein cholesterol levels, such as fibrates and niacin, have not sufficiently reduced adverse outcomes [3]. That emphasizes the need of identifying a medication that targets multiple and additional mechanisms linked to the incidence of CV events and the causative pathway of atherosclerosis [10].

Icosapent ethyl (IPE) has gained significant recognition as an additive agent recently. IPE is an ethyl ester of the omega-3 polyunsaturated fatty acid eicosapentaenoic acid (EPA) that works in the manner of a plasma-binding protein in the blood [11]. Through its effects on the onset of plaque development and plaque rupture, EPA plays a beneficial role in the pathophysiologic cascade of plaque development [12]. A number of specific salutary actions on atherosclerotic plaque factors have been reported including antioxidant effects, anti-inflammatory effects, decreased macrophage accumulation, improved endothelial function, decreased foam cell accumulation, decreased adhesion of monocytes, increased fibrous-cap thickness, and an increase in resolvins, a class of pro-resolving lipid mediators [10,13,14]. EPA is scientifically plausible as a potential anti-atherosclerotic agent based on mechanistic, pathophysiologic, outcomes, and plaque-imaging studies [12].

The IPE compound within the drug known as Vascepa has been shown to decrease the plasma level of atherogenic parameters such as TGs, non-high-density lipoprotein cholesterol (non-HDL-C), oxidized low-density lipoprotein particles (ox-LDL), and remnant cholesterol remnant-like particle cholesterol (RLP-C) without raising low-density lipoprotein cholesterol (LDL-C) levels in patients with very high TG levels and statin-treated patients with high TG levels and high residual CV risk [15,10,13]. Significant reductions in important ischemic events were reported with IPE in the international, double-blind, randomized, placebo-controlled Reduction of Cardiovascular Events with Icosapent Ethyl Intervention Trial (REDUCE-IT) study [16]. The primary endpoint (composed of CV death, nonfatal myocardial infarction, nonfatal stroke, coronary revascularization, or unstable angina requiring hospitalization), the important secondary endpoint (composed of CV death, nonfatal myocardial infarction, or nonfatal stroke), and individual components of those endpoints all had lower incidences following IPE treatment [16,17]. Since the publication of REDUCE-IT, the usage of IPE has been recommended by the American Diabetes Association (ADA), National Lipid Association (NLA), and the European Society of Cardiology (ESC)/European Atherosclerosis Society (EAS), to further decrease ASCVD risk in selected patients [18]. In this systematic review, we will summarize the clinical benefits of IPE and its role in CV risk reduction.

## Review

Methods

The Preferred Reporting Items for Systematic Reviews and Meta-analysis (PRISMA) criteria were meticulously adhered to when conducting the literature searches and compiling the findings [19]. A thorough search was performed on PubMed, PubMed Central (PMC), and Google Scholar databases to obtain the information required, and these databases were systematically searched up to July 28, 2022. Additional studies were also looked for in the reference list of the papers that were found during the search.

Search Strategy and Selection Criteria

The pre-determined inclusion criteria were Published as Randomized Control Trials, Clinical Trials, Reviews, Systematic Reviews, Meta-Analysis, and Free-full text. Due to the large number of articles, only the first 400 articles from Google Scholar were assessed. Zotero (Reference Manager) was used to import and manage all references. Based on the database used, the keywords from past literature and Medical Subject Headings (MeSH) were used to select the field search used in the procedure, as shown in Table 1.

**Table 1:** The Method of Conducting a Bibliographic Search in the Databases Using the Appropriate Filters PMC - PubMed Central, CAD - coronary artery disease

Databases	Keywords	Search Strategy	Filters	Search Results
PubMed	Icosapent Ethyl OR Vascepa OR Ethyl eicosapentaenoic acid OR eicosapentaenoic acid ethyl ester OR coronary artery disease OR CAD OR Myocardial ischemia OR myocardial infarction OR cardiovascular risk reduction	#1: “Icosapent Ethyl” [tw] OR Vascepa [tw] OR “Ethyl eicosapentaenoic acid” [tw] OR "eicosapentaenoic acid ethyl ester" [Supplementary Concept] #2: coronary artery disease OR CAD OR ("Coronary Artery Disease/drug therapy" [Majr] OR "Coronary Artery Disease/prevention and control" [Majr] OR "Coronary Artery Disease/therapy" [Majr]) OR “Myocardial ischemia” [tw] OR “myocardial infarction” [tw] OR cardiovascular risk reduction. #1 AND #2 - 25	Free full text, Clinical Trial, Meta-Analysis, Randomized Control Trial, Review, Systematic Review, Humans	25
PMC	Icosapent Ethyl OR Vascepa OR Ethyl eicosapentaenoic acid OR eicosapentaenoic acid ethyl ester OR coronary artery disease OR CAD OR Myocardial ischemia OR myocardial infarction OR cardiovascular risk reduction	#1: “Icosapent Ethyl” [tw] OR Vascepa [tw] OR “Ethyl eicosapentaenoic acid” [tw] OR "eicosapentaenoic acid ethyl ester" [Supplementary Concept] #2: coronary artery disease OR CAD OR ("Coronary Artery Disease/drug therapy" [Majr] OR "Coronary Artery Disease/prevention and control" [Majr] OR "Coronary Artery Disease/therapy" [Majr]) OR “Myocardial ischemia” [tw] OR “myocardial infarction” [tw] OR cardiovascular risk reduction. #1 AND #2 - 984	Open access	741
Google Scholar	Icosapent Ethyl, Coronary artery disease	Icosapent ethyl and coronary artery disease - 4240	Since 2018	2700

Study Results and Bias Assessment

**Figure 1:**
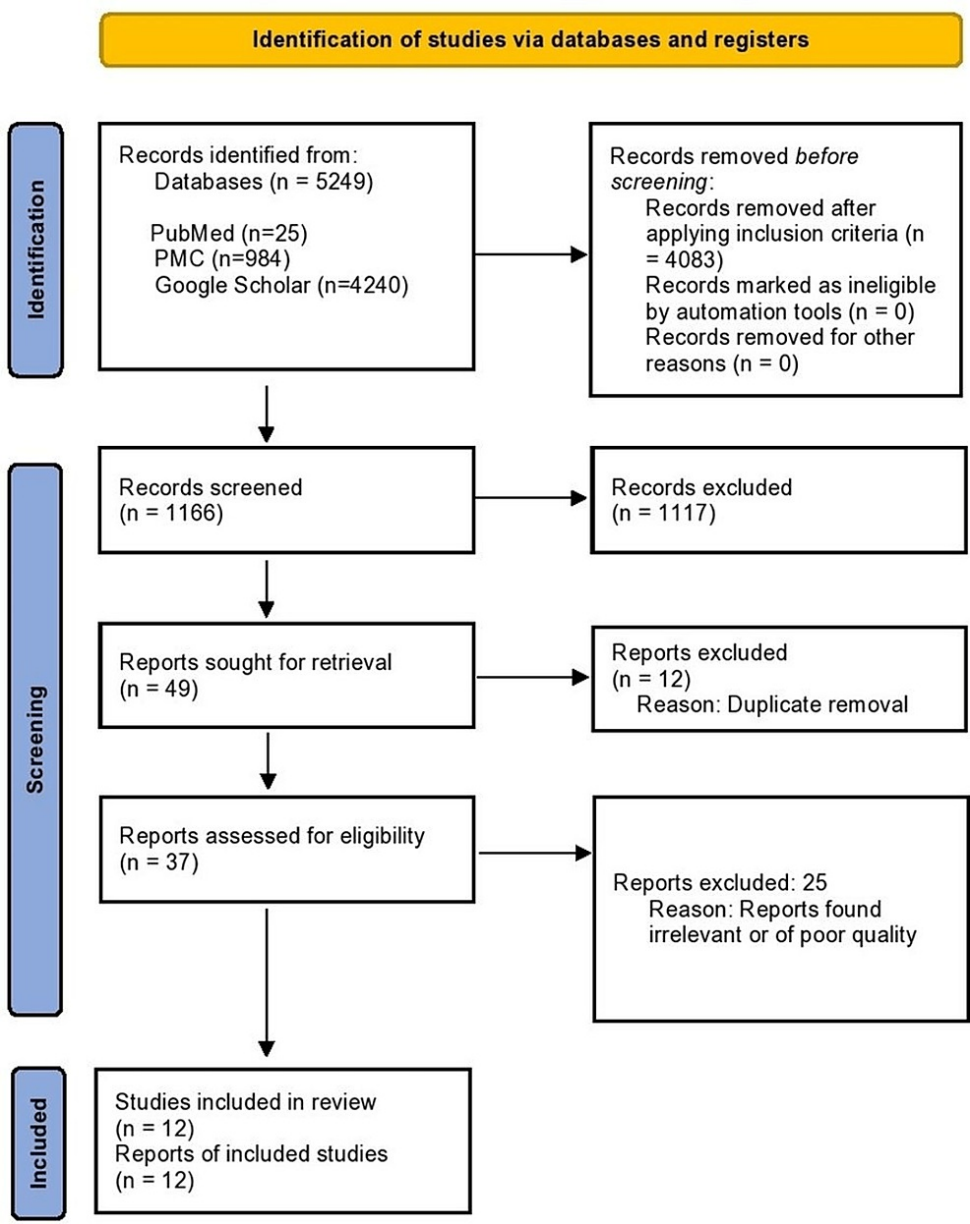
PRISMA 2020 Flow Diagram for Systematic Reviews PMC - PubMed Central, PRISMA - Preferred Reporting Items for Systematic Reviews and Meta-Analysis

In the initial search, 5249 publications were found. After applying inclusion criteria to these, 4083 articles were eliminated. After evaluating titles and abstracts for 1166 publications, another 1117 were eliminated. Consequently, 49 papers were retrieved with the full text available. After the removal of duplicates, 37 articles were reviewed and 12 articles (One Non-Randomized Clinical Trial, Three Systematic Reviews, Three Randomized Controlled Trials, and Five Reviews) were included in the review. A PRISMA flow diagram illustrating the screening procedure and study selection is shown in Figure 1 [19].

Risk of Bias in Individual Studies

The following tools were used to assess the quality and bias of all remaining full articles based on the type of study: Cochrane Collaboration’s risk of bias tool (CCRBT) for Randomized Controlled Trials (RCT) [2], Newcastle Ottawa tool (NOS) for Non-Randomized Clinical Trials (NRCT) [3], Assessment of Multiple Systematic Reviews 2 (AMSTAR 2) checklist for Systematic Review (SR) [4], and Scale for the Assessment of Narrative Review Articles 2 (SANRA 2) checklist for narrative reviews [5]. The quality of the final papers selected was also verified by a second author to decrease the risk of bias. Each assessment instrument required a score of at least 70% to be acceptable (Table 2).

**Table 2:** Quality Assessment of Each Study CCRBT - Cochrane Collaboration’s risk of bias tool, RCT - Randomized Controlled Trials, NOS - Newcastle Ottawa tool, AMSTAR 2 - Assessment of Multiple Systematic Reviews 2, RoB - Risk of Bias, SANRA 2 - Scale for the Assessment of Narrative Review Articles 2, PICO - Patient/Population, Intervention, Comparison and Outcomes

Quality Assessment Tool	Type of Study	Items and Their Characteristics	Total Score	Accepted Score (>70%)	Accepted Studies
CCRBT [20]	RCTs	Seven items: random sequence generation and allocation concealment (selection bias), selective outcome reporting (reporting bias), other sources of bias, blinding of participants and personnel (performance bias), blinding of outcome assessment (detection bias), and incomplete outcome data (attrition bias). Bias assessed as Low risk, High risk, or Unclear.	7	5	Budoff et al. [15], Gaba et al. [21], Verma et al. [22]
NOS [23]	Non-Randomized Control Trials	Eight items: (1) Representativeness of the exposed cohort, (2) Selection of the non-exposed cohort, (3) Ascertainment of exposure, (4) Demonstration that an outcome of interest was not present at the start of the study, (5) Comparability of cohorts on the basis of the design or analysis*, (6) Assessment of outcome, (7) Was follow-up long enough for outcomes to occur, (8) Adequacy of follow-up of cohorts. Scoring was done by placing a point on each category. Scored as 0, 1, 2. * Maximum of two points are allotted in this category.	9	7	Kurita et al. [24]
AMSTAR 2 [25]	Systematic reviews	Sixteen items: (1) Did the research questions and inclusion criteria for the review include the components of PICO? (2) Did the report of the review contain an explicit statement that the review methods were established prior to the conduct of the review, and did the report justify any significant deviations from the protocol? (3) Did the review authors explain their selection of the study designs for inclusion in the review? (4) Did the review authors use a comprehensive literature search strategy? (5) Did the review authors perform study selection in duplicate? (6) Did the review authors perform data extraction in duplicate? (7) Did the review authors provide a list of excluded studies and justify the exclusions? (8) Did the authors describe the included studies adequately? (9) Did the review authors use a satisfactory technique for assessing the risk of bias (RoB) in individual studies that were included in the review? (10) Did the review authors report on the sources of funding for the studies included in the review? (11) If meta-analysis was justified, did the review authors use appropriate methods for the statistical combination of results? (12) If meta-analysis was performed, did the review authors assess the potential impact of RoB in individual studies on the results of the meta-analysis or other evidence synthesis? (13) Did the review authors account for RoB in individual studies when interpreting/ discussing the results of the review? (14) Did the review authors provide a satisfactory explanation for, and discussion of, any heterogeneity observed in the results of the review? (15) If they performed quantitative synthesis did the review authors carry out an adequate investigation of publication bias (small study bias) and discuss its likely impact on the results of the review? (16) Did the review authors report any potential sources of conflict of interest, including any funding they received for conducting the review? Scored as YES or NO. Partial Yes was considered as a point.	16	12	Thakur et al. [11], Khan et al. [26], Sekikawa et al. [27]
SANRA 2 [28]	Narrative review	Six items: justification of the article’s importance to the readership, statement of concrete aims or formulation of questions, description of the literature search, referencing, scientific reason, and appropriate presentation of data. Scored as 0, 1 or 2.	12	9	Pareek et al. [3], Gaba et al. [29], Nelson et al. [10], Jia et al. [18], Wang et al. [5]

Results

Table 3 describes the key characteristics of clinical trials, observational studies, and reviews included in this study.

**Table 3:** Main Characteristics of the Selected Studies RCT - Randomized Control Trials, TG -Triglycerides, LDL-C - Low-Density Lipoprotein-C, LAP - Low Attenuation Plaque, IPE - Icosapent Ethyl, EVAPORATE - Effect of Vascepa on Improving Coronary Atherosclerosis in People with High Triglycerides Taking Statin Therapy, CV - Cardiovascular, MI - Myocardial Infarction, HR - Hazard Ratio, CI - Confidence Interval, ARR - Absolute Risk Reduction, CABG - coronary artery bypass grafting, EPA - Eicosapentaenoic Acid, PCI - Percutaneous Coronary Intervention, IMR - Index of Microcirculatory Resistance, TnT - Troponin T, CK-MB - Creatine Kinase-MB, DHA - Docosahexaenoic acid, FA - Fatty Acid, ASCVD - Atherosclerotic Cardiovascular Disease, OM3 - Marine Omega-3 Fatty Acid, REDUCE-IT - Reduction of Cardiovascular Events with Icosapent Ethyl Intervention Trial, MDCT - Multidetector Computed Tomography, TP - Total Plaque, TNCP - Total Non-Calcified Plaque, FF - Fibrofatty, F - Fibrous, C - Calcified, PCSK9 - Proprotein Convertase Subtilisin/Kexin-Type 9

Author and Year of Publication	Study Type	Patient Criteria	Outcome	Results	Conclusion
1. Budoff et al. 2020 [15]	double-blind, placebo-controlled RCT	Eligibility Criteria: Included patients between 30 and 85 years of age with coronary atherosclerosis (narrowing of ≥20% in one coronary artery by either invasive angiography or multidetector computed tomography angiography) and elevated fasting TG levels (200–499 mg/dL) and LDL-C levels ≥40 and ≤115 mg/dL, patients on statin therapy (with or without ezetimibe) as well as patients on stable diet and exercise for ≥4 weeks prior to study entry. In the trial, assuming a 15% dropout rate, a total of 80 (40 per arm) patients were enrolled. They were randomly assigned to Icosapent ethyl 4 g/d or placebo in a 1:1 fashion, followed after ≥9 months or up to 18 months (if efficacy is not achieved at 9 months).	Primary endpoint: Change in LAP volume measured by multidetector computed tomography (MDCT) angiography [then sequentially: total plaque (TP), total non-calcified plaque (TNCP), fibrofatty (FF), fibrous (F), and calcified plaque (C)].	Change in LAP between the 18-month scan and baseline scan was significantly reduced with IPE compared with placebo (-0.3 ± 1.5 vs. 0.9 ± 1.7 mm3, P = 0.006). Other parameters of interest (in order of pre-specified analysis) include total plaque (-9% with IPE vs. +11% with placebo, P = 0.002), total non-calcified plaque (-19% vs. +9%, P = 0.0005), fibrofatty (-34% vs. +32%, P = 0.0002), fibrous (-20% vs. 1%, P = 0.003), and calcified plaque (-1% vs. +15%, P = 0.053).	The outcomes of EVAPORATE study reveal that Icosapent ethyl induces slowing of plaque progression when added to statin therapy.
2. Gaba et al. 2021 [21]	Phase IIIb double-blind, placebo-controlled RCT	Eligibility Criteria: Age ≥45 years with established cardiovascular disease or Age ≥50 years with diabetes mellitus and at least one additional risk factor, fasting TG level of 150-499 mg/ dL(1.69-5.63 mmol/L) and an LDL cholesterol level of 41-100 mg/dL(1.06-2.59 mmol/L) and had been receiving a stable dose of a statin for at least four weeks. In the trial, a total of 8179 patients were enrolled, and they were assigned to IPE 4 g/dL or placebo and were followed for a median of 4.9 years.	Primary endpoint: composite of CV death, nonfatal MI (including silent MI), nonfatal stroke, coronary revascularization, or unstable angina requiring hospitalization. Secondary endpoint: composite of CV death, nonfatal MI, and nonfatal stroke. Other endpoints: individual components of the primary and secondary composite endpoints, specifically CV death, MI, stroke, coronary revascularization, and hospitalization for unstable angina.	Comparing IPE with placebo, the rate of occurrence of primary composite endpoint was 19.1% vs. 24.6% (HR: 0.74 [95% CI: 0.67-0.81]; P < 0.0001), ARR with IPE is 6.2% (95% CI, 2.3%–10.2%) and that of secondary composite endpoint was 10.5% vs. 13.6% (HR: 0.75 [95% CI: 0.66-0.85]; P < 0.0001), ARR with IPE is 6.0% (95% CI, 2.5%–9.5%).	Among patients with elevated triglyceride levels despite the use of statins, the risk of CV events, including MI, coronary revascularizations, and cardiovascular death was significantly lower among those who received 4 g/dl of IPE than among those who received placebo.
3. Verma et al. 2021 [22]	A multicenter, placebo-controlled, double-blind trial on a subgroup of patients from the REDUCE-IT trial with a history of CABG.	Eligibility Criteria: Age ≥45 years with established cardiovascular disease or Age ≥50 years with diabetes mellitus and at least one additional risk factor, fasting TG level of 150-499 mg/dL (1.69-5.63/L) and an LDL cholesterol level of 41-100 mg/dL (1.06-2.59 mmol/L), had been receiving a stable dose of a statin for at least four weeks, and previous history of CABG. Out of 1837 patients, 897 patients were randomized to icosapent ethyl 4 g/dL and 940 to placebo and were followed for a median of 4.8 years.	Primary endpoint: composite of CV death, nonfatal MI (including silent MI), nonfatal stroke, coronary revascularization, or unstable angina requiring hospitalization. Secondary endpoint: composite of CV death, nonfatal MI, and nonfatal stroke.	Comparing IPE with placebo, the rate of occurrence of primary composite endpoint was 22.0% vs. 28.2% (HR, 0.76 [95% CI, 0.63–0.92]; P=0.004) and that of secondary composite endpoint was 14.7% vs. 20.7% (HR, 0.69 [95% CI, 0.56-0.87]; P=0.001).	Among patients with a previous history of CABG, significant relative and absolute risk reductions in primary and recurrent CV events are seen with IPE treatment, as compared to placebo.
4. Kurita et al. 2014 [24]	A prospectively planned non-randomized clinical trial	Eligible patients: patients who were scheduled to undergo an elective and de novo stent implantation in the native coronary artery for stable angina pectoris. A total of 178 patients were enrolled in this trial. Out of them, 89 patients received oral EPA 1800 mg/day in addition to stain therapy, and 89 patients received only statin therapy one month prior to elective PCI.	Peri-procedural cardiac biomarkers and Post-procedural IMR	IMR: significantly decreased in the EPA group compared to the control group [19.8 (6.4, 51.1) vs. 27.8 (8.2, 89.3), p = 0.003]. Cardiac biomarkers: Treatment with EPA significantly reduced the change from baseline in TnT [(0.0 (0.0, 0.5) vs. 0.0 (0.0, 1.4), p = 0.01] compared with controls, while there were no significant differences in CK-MB between both groups.	The incidence of type IVa (peri-procedural) MI can be markedly reduced by pre-treatment with EPA in addition to statin compared to statin therapy only, in patients undergoing PCI.
5. Thakur et al. 2020 [11]	A systematic literature review evaluating the association between high triglycerides and low-density lipoproteins and prevalence of cardiovascular diseases and the role of IPE on cardiovascular risk reduction	The Research Gate, National Center for Biotechnology Information, PubMed, and Google Scholar databases were thoroughly searched to find relevant peer-reviewed articles from 2008-2020. Other sources, such as school newsletters and the World Health Organization, were also searched to identify peer-reviewed articles. All English-language data were examined and articles with small sample sizes and articles focusing on multiple comorbidities were excluded.			The common factor in the occurrence of cardiovascular illnesses and diabetes is triglycerides which are targeted by Icosapent ethyl. The drug significantly lowers the available triglycerides, with minor or no significant changes in lipoprotein levels which makes Icosapent ethyl the most effective medication for the prevention of cardiovascular diseases.
6. Khan et al. 2021 [26]	A systematic review and meta-analysis of 38 RCT	The phrases “omega-3 fatty acid,” “eicosapentaenoic acid,” “docosahexaenoic acid,” “fish oil,” “cholesterol,” “triglycerides,” and “cardiovascular disease” were used as search terms in electronic databases of EMBASE, PubMed, ClinicalTrials.gov, and Cochrane library, through June 7, 2021. The pre-determined criteria used by the author were (1) randomized controlled trials that compared omega-3 FA intake (EPA or EPA+DHA) vs. control (placebo, no supplementation, or lower dose of omega-3 FA) in adults; (2) follow-up duration of at least 12 months; and (3) trials must report mortality and cardiovascular outcomes of interest.			Moderate certainty of evidence supporting the role of omega-3 FAs in the reduction of cardiovascular mortality and outcomes was noted in this systematic review and meta-analysis. This meta-analysis gives confidence about the function of omega-3 fatty acids, specifically EPA, in the current ASCVD residual cardiovascular risk reduction treatment strategy.
7. Sekikawa et al. 2019 [27]	Systematic review and meta-analysis of six RCTs demonstrating the role of marine omega-3 fatty acids supplementation in slowing the progression of atherosclerosis	Using PubMed, Embase, Cochran Central Register of Controlled Trials, and clinicaltrials.gov, a systematic search was performed from the earliest publication date to March 1, 2019. The inclusion criteria were RCTs (1) conducted among adults (≥18 years) without hemodialysis, (2) using high-dose OM3 supplements (defined as ≥3 g/day of OM3 or ≥1.8 g/day of OM3 in Japan) with the purity of OM3 ≥90% as the intervention, (3) using atherosclerosis as the primary outcome, (4) reporting percent or absolute change of atherosclerosis, (5) with the intervention period ≥6 months, and (6) with articles published and available in full-text English language.			By its anti-atherosclerotic properties, High-dose OM3 slows the progression of atherosclerosis.
8. Pareek et al. 2021 [3]	Narrative review	In this review, the author has presented a summary of recent meta-analysis findings, major observational studies, and a full discussion of landmark trials, such as REDUCE-IT that provides an overview of evidence and recommendations related to triglyceride-lowering therapy in the primary and secondary preventive settings.			Given the expanding population of patients with hypertriglyceridemia and other risk factors, IPE will have an increasingly major impact on cardiovascular risk reduction. As compared to more expensive therapies such as the proprotein convertase subtilisin/kexin type 9 (PCSK9) inhibitors, alirocumab, and evolocumab, IPE will be very favorable in terms of cost-effectiveness in both primary and secondary prevention.
9. Gaba et al. 2022 [29]	A Narrative review summarizing the key findings of REDUCE-IT				IPE reduced the incidence of clinically important ischemic events, including cardiovascular death, nonfatal stroke, nonfatal MI, coronary revascularization, and unstable angina requiring hospitalization, in the REDUCE-IT trial. Subsequent studies have supported these findings and extended the findings to patients with high cardiovascular risk, including those with prior MI, prior coronary revascularization with PCI or CABG, diabetes, and severe renal dysfunction. In light of these findings, all guidelines must be revised and expanded to include IPE as a treatment for patients with high cardiovascular risk and mild or moderate hypertriglyceridemia.
10. Nelson et al. 2017 [10]	Narrative review	In this review, the author has covered the possible impacts of EPA on CV outcomes as well as preclinical and clinical research on the effects of EPA on atherosclerotic plaques.			EPA has been shown to increase the fibrous-cap thickness of plaques and thereby, decrease the chances of plaque rupture. In patients with hypertriglyceridemia, IPE, a high-purity prescription form of EPA ethyl ester, has been shown to reduce triglyceride levels and markers of inflammation.
11. Jia et al. 2020 [18]	Narrative review	In his review, the author has summarized the contemporary evidence of IPE in ASCVD risk reduction with a focus on the clinical implication of this promising therapy.			A confluence of evidence supports the efficacy of IPE in reducing ASCVD risk, culminating in the REDUCE-IT trial. Its inflammation and triglyceride-lowering properties account for the cardio-protective effect of IPE. Despite some unknown facts such as the molecular mechanism of action and cost-effectiveness, the effect of IPE on residual ASCVD risk in high-risk patients already on statin therapy appears promising.
12. Wang et al. 2020 [5]	Narrative review summarizing key clinical trials	The review summarizes findings from key clinical trials for EPA e.g., MARINE and ANCHOR that lend to the landmark clinical trial REDUCE-IT, which demonstrated the beneficial effects of IPE for cardiovascular risk reduction and led to its approval.			Icosapent ethyl, the first drug of its class to be approved, is an effective and safe choice in lowering triglycerides and further lowering CV mortality and morbidity. Though its mechanism of action is unclear, the benefits of icosapent ethyl outweigh the expected level of triglyceride reduction and are most likely due to its anti-inflammatory and antithrombotic effects.

Discussion

The following sections discuss the mechanism of action of EPA, the role of IPE in the management of hypertriglyceridemia and alleviating cardiovascular risk, and the safety of IPE.

Potential Mechanisms of Action of EPA

IPE (Vascepa) is a high-purity prescription form of EPA ethyl ester approved by the United States (US) Food and Drug Administration (FDA) at a dose of 4 g/d as an adjunct to diet. EPA is an omega-3 polyunsaturated fatty acid (OM3) that is incorporated into membrane phospholipids and atherosclerotic plaques [12]. EPA has been shown to inhibit hepatic very low-density lipoprotein (VLDL)-TG synthesis by interfering with the non-esterified fatty acid influx into the liver [18]. By promoting apoB100 degradation and upregulating beta-oxidation of free fatty acids in hepatocytes, omega-3 fatty acids (FAs) are also thought to prevent VLDL assembly and secretion. This leads to a decrease in triglyceride (TG) synthesis as well as an increase in VLDL-TG clearance by upregulation of lipoprotein lipase (LpL) expression in adipose [18]. The effects of EPA and its metabolites on TG levels may be attributed in part to the activation of the peroxisome proliferator-activator receptor alpha (PPAR- α) [3].

Although REDUCE-IT identified high-normal to moderately elevated TGs as a promising therapeutic target for further CV risk reduction, the exact mechanisms remain unclear. They appear to be mediated by EPA and its anti-inflammatory, antithrombotic, and plaque stabilization effects downstream [5]. Omega-3 FAs, particularly EPA, have the ability to incorporate into platelet membranes and replace omega-6 FAs [5]. By competing with omega-6 FAs for cyclooxygenase enzymes, EPA generates thromboxane A3 while lowering the synthesis of thromboxane A2, a strong atherothrombotic agent [29]. In addition, when combined with a statin, EPA increases the endothelial release of nitric oxide, which is a powerful platelet inhibitor [5]. OM3 is a precursor to a group of lipid mediators known as specialized pro-resolving mediators (SPM), which include resolvins, protectins, and maresins and plays an important role in the counter-regulation of inflammation including in atherosclerosis [27]. The reported effects of EPA on endothelial function were also a likely factor in the CV advantages of IPE, as evidenced by studies indicating decreased indicators of endothelial dysfunction after therapy [5]. With EPA incorporated into cell membranes, membrane stability is enhanced even with increasing cholesterol load, resulting in a protective effect against endothelial dysfunction [18]. Moreover, studies have shown that OM3 reduces native T-cell differentiation to type I helper T cells (Th1), which are predominant cells found in atherosclerotic plaques and pro-inflammatory markers of adaptive immunity [27].

Several imaging studies such as coronary computed tomographic angiography (CCTA) and multidetector computed tomography (MDCT) angiography have evidenced that IPE reduces coronary vulnerable plaque burden, confirming its cardio-protective properties. Vulnerable atherosclerotic plaques are those with unique anatomical and biologic characteristics (i.e., thin cap atherofibromas, large necrotic cores, macrophage infiltration, etc.) that put them at risk of rupture and coronary thrombosis [29]. It has been shown that EPA concentrations in atherosclerotic plaques are associated with lower foam cells and decreased expression of matrix metalloproteinases, which are thought to increase plaque stability [18]. In Effect of Vascepa on Improving Coronary Atherosclerosis in People with High Triglycerides Taking Statin Therapy (EVAPORATE) study, patients treated with IPE 4 g/day had early slowing of progression in total plaque and noncalcified plaque volume compared with patients treated with placebo [5]. At 18 months, IPE treatment showed a significant reduction in low attenuation plaque (LAP) volume (-0.3 +/- 1.5 vs. 0.9 +/- 1.7 mm^3^, P = 0.006), a well-established marker of plaque vulnerability [15].

Role of IPE in Management of Hypertriglyceridemia and Alleviation of Cardiovascular Risk

Eicosapentaenoic acid is a type of omega-3 acid FA found in high-fat seafood such as salmon fish [11]. Omega-3 acids are commonly known to protect from heart disease throughout the world [30]. As part of a healthy diet, it significantly reduces coronary heart illness, high blood pressure, and high cholesterol levels [11]. IPE, the form of organic omega-3 FA, has the same effect and ability to function on the regulation of TG levels as well as the decrease in lipoprotein rise. This drug's impact is so practical and thoroughly proven that the US FDA approved it at a dose of 4 g/day to reduce cardiovascular ailments [11].

REDUCE-IT was a phase IIIb, double-blind, placebo-controlled trial by Gaba et al. (published in 2021) that was designed to study the beneficial effects of IPE on investigator-reported events in statin-treated patients with elevated TGs [21]. A total of 8,179 statin-treated patients with controlled low-density lipoprotein cholesterol (LDL-C) and moderately elevated TGs were randomized to 4 g daily of either IPE or matched placebo (two capsules twice daily with food). The primary endpoint was a composite of CV death, nonfatal myocardial infarction (MI) (including silent MI), nonfatal stroke, coronary revascularization, or unstable angina requiring hospitalization. The key secondary endpoint was a composite of CV death, nonfatal MI, and nonfatal stroke. Other endpoints included the individual components of the primary and secondary composite endpoints, specifically CV death, MI, stroke, coronary revascularization, and hospitalization for unstable angina. The blinded investigators on each site collected and reported potential endpoints, and they were subsequently adjudicated by a blinded Clinical Endpoint Committee (CEC) according to a predetermined charter. The median follow-up time was 4.9 years (IQR: 3.5-5.3 years) [21].

In the trial, the primary endpoint was reported in 1,790 patients (782 (19.1%) treated with IPE and 1,008 (24.6%) treated with placebo, hazard ratio (HR): 0.74; 95% Confidence Interval (CI): 0.67-0.81; p < 0.0001) in the investigator-reported group, it occurred in 1,606 patients (705 (17.2%) treated with IPE and 901 (22.0%) treated with placebo, HR: 0.75; 95% CI: 0.68-0.83; p < 0.0001) in the adjudicated group. This conclusion was primarily driven by a larger number of hospitalizations for unstable angina recorded in the investigator-reported group that was not validated by adjudicators. However, similar HRs and narrow CI indicate that the direction of treatment effect was similar between the two groups. The secondary endpoint occurred in 987 patients (430 (10.5%) treated with IPE and 557 (13.6%) treated with placebo, HR: 0.75; 95% CI: 0.66-0.85; p < 0.0001)) in the investigator-reported group and in 1,065 patients (459 (11.2%) treated with IPE and 606 (14.8%) treated with placebo, HR: 0.74; 95% CI: 0.65-0.83; p < 0.0001) in the adjudicated group. This finding was mostly driven by a decreased number of MIs reported by investigators, particularly the inconsistent identification of silent MIs. However, as noted with the primary endpoint, the degree of treatment effect was similar between the investigator-reported or adjudicated group. Overall, in the novel analysis of REDUCE-IT, the author found that the majority of adverse CV events among patients treated with statins who have controlled LDL-C and moderately elevated TGs were significantly reduced with IPE, regardless of whether they were assessed by blinded site investigators or central, blinded adjudication [21].

In the narrative review of key findings from the REDUCE-IT by Gaba et al. (published in 2022) [29], the author has summarized the extended findings from the REDUCE-IT trial in patients with high cardiovascular risk. In the patients with prior, there was a significant reduction in primary composite events from 26.1% to 20.2% (HR 0.74, 95% CI 0.65-0.85, p = 0.00001) in patients treated with IPE compared to patients treated with placebo. In REDUCE-IT REVASC, the incidence of requiring revascularization with either percutaneous coronary intervention (PCI) or coronary artery bypass grafting (CABG) was evaluated among patients treated with IPE. There was a 34% reduction in first coronary revascularizations among patients treated with IPE vs. placebo (9.2% vs. 13.3%, HR 0.66, 95% CI 0.58-0.76, p < 0.0001). According to REDUCE-IT CABG, primary composite endpoint events in the patients with prior history of CABG were reduced by 24% in those treated with IPE compared with placebo (HR 0.76, 95% CI 0.63-0.92, p = 0.004). In the individuals with chronic kidney disease (estimated Glomerular Filtration Rate <60 mL/min/1.73 m^2^), there was a 29% relative and 7.1% absolute risk reduction (ARR) (27.2% vs. 35.6%, HR: 0.71, 95% CI: 0.59-0.85) in the primary composite endpoint when compared to placebo-treated patients. According to REDUCE-IT DIABETES, the incidence of primary endpoint events was decreased from 22.4% to 18.1% (HR 0.77, 95% CI 0.68-0.87) in IPE-treated diabetic patients. Subsequent analysis on REDUCE-IT has strengthened its findings, in both the investigator-reported and adjudicated subgroups, and expanded the findings to individuals with high cardiovascular risk and concluded that IPE is proved to be effective in cardiovascular risk reduction at a dose of 4 g/day. In sum, these results demand that all international guidelines be reviewed and expanded to include IPE as a treatment option for patients with high cardiovascular risk and mild to moderate hypertriglyceridemia [29].

In the analysis by Verma et al. (published in 2021) on the subgroup of patients from the REDUCE-IT trial with a history of CABG, 1837 patients with a history of CABG were included [22]. Out of them, 897 were randomized to IPE and 940 to placebo. Patients with a history of CABG had a total of 462 positively adjudicated primary events; in particular, 22.0% of patients randomized to IPE and 28.2% of patients randomized to placebo had a primary endpoint event (HR, 0.76 (95% CI, 0.63-0.92); p = 0.004). IPE therapy is linked to significant relative and ARRs with regard to the first and recurrent ischemic episodes in patients with a history of CABG when compared to placebo. In light of these findings, IPE should be taken into account as a crucial adjuvant therapy for the subsequent prevention of adverse cardiac outcomes in patients post-CABG [22].

EVAPORATE was a phase IIIb, double-blind, placebo-controlled trial by Budoff et al. (published in 2020) that was designed to study whether IPE 4 g/day along with diet and statin therapy could result in greater changes from baseline in plaque volume, measured with serial MDCT, than placebo in statin-treated patients [15]. A total of 80 patients were randomized 1:1 to IPE or placebo to evaluate progression rates of plaque volume on CCTA. Participants underwent an MDCT scan at baseline and then an interim scan at nine months and the final MDCT scan at 18 months. Changes in LAP between the baseline scan and the 18-month scan, the study's primary endpoint, were markedly reduced by IPE in comparison to placebo (-0.3 ± 1.5 vs. 0.9 ± 1.7 mm^3^, p = 0.006). With the exception of calcified plaque, the final data at 18 months demonstrated a significant improvement in all plaque volumes using 4 g/day of IPE in comparison to placebo. Despite lowering atherosclerotic plaque, statin therapy increases coronary calcification. However, according to this study, IPE showed no increase in coronary artery calcium volume, with a trend of decreasing calcification compared with placebo (p = 0.053). Since LAP is linked to vulnerability and potential MI, removing this necrotic core with IPE in EVAPORATE trial is highly supportive of the clinical findings from the Japan EPA Lipid Intervention Study (JELIS) and REDUCE-IT and consistent with the Combination Therapy of Eicosapentaenoic acid and Pitavastatin vs. Pitavastatin Alone for Coronary Plaque Regression Evaluated by Intravascular Ultrasonography (CHERRY) and other studies that show EPA is linked to slower plaque progression, plaque stabilization, and decreased ASCVD events. In conclusion, the EVAPORATE results give significant mechanistic information on the therapeutic effects of IPE on plaque features and vulnerability by showing the potential to slow the advancement of atherosclerosis and induce its reversal [15].

In a prospectively planned NRCT by Kurita et al. (published in 2014) [24], the effect of EPA on periprocedural (type IVa) MI following elective PCI has been assessed. In this study, 178 consecutive patients with stable angina pectoris who underwent elective surgery and de novo stent implantation were treated prospectively with statins or statins and EPA 900 mg twice a day (BID) for at least one month before PCI. Ultimately, 165 patients with 165 de novo stent implantations (82 patients in the EPA group and 83 in the control group) who fulfilled the inclusion criteria were included in the data analysis. The blood sample was collected before and 16-20 h after the procedure to measure cardiac biomarker levels. Aspirin was given to every patient 24 hours before surgery. In order to prevent coronary spasms, patients received a 0.5 mg intracoronary dose of isosorbide dinitrate before undergoing coronary angiography and PCI. After PCI, the index of microcirculatory resistance (IMR) was measured using the previously described technique [31-33]. While the change from baseline in Creatine Kinase-MB (CK-MB) was similar in both groups, the change from baseline in Troponin-T (TnT) was considerably lower in the EPA group compared to the control group (0.0(0.0, 0.5) vs. 0.0(0.0, 1.4), p = 0.01). Just after PCI, IMR was randomly measured in 30 patients with EPA treatment and 32 controls, and it was significantly lower in the EPA group (19.8 (6.4, 51.1) vs. 27.8 (8.2, 89.3), p = 0.003). In comparison to continuous statin therapy alone, pre-treatment with EPA dramatically decreased peri-procedural TnT levels and the incidence of type IVa MI. This effect of EPA may be partly related to its capacity to lessen PCI-induced microvascular dysfunction [24].

In the SR by Khan et al. (published in 2021) [26], a meta-analysis of 38 RCTs was conducted to demonstrate the effect of omega-3 FAs on cardiovascular mortality and outcomes. In this meta-analysis of 38 trials comprising 149,051 adult participants, trials of EPA showed higher relative reductions in cardiovascular outcomes than those of Eicosapentaenoic Acid + Docosahexaenoic acid (EPA + DHA), with significant interaction terms (HR, 0.91 (95% CI, 0.87-0.96); p =< 0.01). In EPA trials compared with EPA + DHA trials, relative reductions were substantial, suggesting differential effects of EPA and DHA on cardiovascular risk reduction. Though two recent negative trials evaluating EPA + DHA in the prevention of ASCVD have created some confusion in the scientific community, The role of omega-3 FAs, notably EPA, in the current ASCVD residual cardiovascular risk reduction treatment strategy is reassured by this meta-analysis [26].

In the SR by Sekikawa et al. (published in 2019) [27], a meta-analysis of six RCTs was performed to demonstrate the effect of high-dose marine omega-3 FA supplementation on the progression of atherosclerosis. A total of 693 participants from six RCTs were included after considering the inclusion criteria. In this analysis, even after the Bonferroni correction (study location, site of atherosclerosis, use of placebo, statin use, presence of coronary heart disease, and source of OM3), the impact of high doses of OM3 was substantially different in six of seven categories. Therefore, it is unclear if the impact of high doses of OM3 varies in proportion to these parameters. But given that both JELIS (1.8 g/day of EPA) in Japan and REDUCE-IT (4.0 g/day of IPE (3.84 g/day of EPA) largely in Western countries demonstrated significant relative risk reduction in cardiovascular disease (CVD) outcomes, it seems doubtful that the effect varies by study location. Based on the findings of JELIS and REDUCE-IT, high-dose OM3 is regarded as adjunctive therapy to statins, which are the gold standard for both secondary and primary prevention of CVD. Whether high-dose OM3 reduces CVD outcomes without statin treatment remains to be answered [27].

Safety of IPE

In JELIS, adverse events were more frequent in the EPA group than in the control group throughout the study (25.3% vs. 21.7%, p = 0.0001), including greater rates of abnormal laboratory results, gastrointestinal problems, dermatologic side effects, and hemorrhage [18]. On the other hand, in REDUCE-IT, major adverse events were seen to be comparable between IPE and placebo (30.6% vs. 30.7%, p = 0.98) [34]. Also, in accordance with the full study population in REDUCE-IT, the rate of atrial fibrillation (AF) was considerably higher when IPE was used (3.1% vs. 2.1%, p = 0.004) [18]. In the patients with a previous history of CABG, the rate of adverse events such as AF or flutter requiring hospitalization for at least 24 hours was higher in IPE-treated patients (5.0% vs. 3.1%; p = 0.03) [22]. These patients also had a higher incidence of bleeding-related adverse events with IPE (2.7% vs. 2.1%, p = 0.06) [8]. While there was no difference between the IPE and placebo groups in terms of new heart failure or heart failure hospitalization [18].

Limitations

There are some limitations to our analysis, the minimal number of publications from only four databases were included and grey literature and other databases are excluded and the evaluation of solely free full-text papers was done. This study's observational approach limits it to showing correlations between variables, but not causal or temporal relationships. More studies are required to find out causal or temporal relationships. The results of our analysis should be evaluated with caution due to the substantial level of variability among the studies that were used.

## Conclusions

IPE, a novel FDA-approved agent, has been proven effective in significantly lowering TG levels and reducing CV risks in patients who are already on optimal statin therapy. Although its mechanisms have yet to be fully explained, IPE, being the first drug of its class to be approved, is believed to exert its cardioprotective effect through its anti-inflammatory and anti-thrombotic properties. From a deeper comprehension of the chemical mechanism of action to practical application and cost-effectiveness, there is still much to learn about IPE. In summary, we herein support the consideration of IPE as an important adjunct therapy for the reduction of residual ASCVD risk in high-risk patients already on statin therapy.
